# Clinical and genetic analysis of spinocerebellar ataxia type 7 (SCA7) in Zambian families

**DOI:** 10.1186/s40673-017-0075-5

**Published:** 2017-11-29

**Authors:** Masharip Atadzhanov, Danielle C. Smith, Mwila H. Mwaba, Omar K. Siddiqi, Alan Bryer, L. Jacquie Greenberg

**Affiliations:** 1 Department of Internal Medicine, University of Zambia School of Medicine, Lusaka, Zambia; 2 Division of Human Genetics, Department of Pathology, Institute of Infectious Disease and Molecular Medicine, Faculty of Health Sciences, University of Cape Town, Cape Town, South Africa; 3 Division of Neurology, Department of Medicine, Faculty of Health Sciences, University of Cape Town, Groote Schuur Hospital, Cape Town, South Africa; 4 Global Neurology Program, Division of Neuro-Immunology, Center for Virology and Vaccine Research, Department of Neurology, Beth Deaconess Medical Center, Harvard Medical School, Boston, MA USA

**Keywords:** Spinocerebellar ataxias, Zambia, SCA7, Macular degeneration

## Abstract

**Background:**

To date, 43 types of Spinocerebellar Ataxias (SCAs) have been identified. A subset of the SCAs are caused by the pathogenic expansion of a CAG repeat tract within the corresponding gene. Ethnic and geographic differences are evident in the prevalence of the autosomal dominant SCAs. Few descriptions of the clinical phenotype and molecular genetics of the SCAs are available from the African continent. Established studies mostly concern the South African populations, where there is a high frequency of SCA1, SCA2 and SCA7. The SCA7 mutation in South Africa (SA) has been found almost exclusively in families of indigenous Black African ethnic origin.

**Objective:**

To present the results of the first clinical description of seven Zambian families presenting with autosomal dominant SCA, as well as the downstream molecular genetic analysis of a subset of these families.

**Methods:**

The study was undertaken at the University Teaching Hospital in Lusaka, Zambia. Ataxia was quantified with the Brief Ataxia Rating Scale derived from the modified international ataxia rating scale. Molecular genetic testing for 5 types of SCA (SCA1, SCA2, SCA3, SCA6 and SCA7) was performed at the National Health Laboratory Service at Groote Schuur Hospital and the Division of Human Genetics, University of Cape Town, SA. The clinical and radiological features were evaluated in seven families with autosomal dominant cerebellar ataxia. Molecular genetic analysis was completed on individuals representing three of the seven families.

**Results:**

All affected families were ethnic Zambians from various tribes, originating from three different regions of the country (Eastern, Western and Central province). Thirty-four individuals from four families had phenotypic features of SCA7. SCA7 was confirmed by molecular testing in 10 individuals from 3 of these families. The age of onset of the disease varied from 12 to 59 years. The most prominent phenotypic features in these families were gait and limb ataxia, dysarthria, visual loss, ptosis, ophthalmoparesis/ophthalmoplegia, pyramidal tract signs, and dementia. Affected members of the SCA7 families had progressive macular degeneration and cerebellar atrophy. All families displayed marked anticipation of age at onset and rate of symptom progression. The pathogenic SCA7 CAG repeat ranges varied from 47 to 56 repeats. Three additional families were found to have clinical phenotypes associated with autosomal dominant SCA, however, DNA was not available for molecular confirmation. The age of onset of the disease in these families varied from 19 to 53 years. The most common clinical picture in these families included a combination of cerebellar symptoms with slow saccadic eye movements, peripheral neuropathy, dementia and tremor.

**Conclusion:**

SCA is prevalent in ethnic Zambian families. The SCA7 families in this report had similar clinical presentations to families described in other African countries. In all families, the disease had an autosomal dominant pattern of inheritance across multiple generations. All families displayed anticipation of both age of onset and the rate of disease progression. Further clinical and molecular investigations of the inherited ataxias in a larger cohort of patients is important to understand the natural history and origin of SCAs in the Zambian population.

## Background

African populations are characterized by high levels of genetic and geographic diversity, and extensive population substructure [1]. Studies of the genetic basis of neurological diseases in African populations can improve our understanding of novel mechanisms of disease pathogenesis and the origins of pathogenic mutations. However, there is very little data on the clinical spectrum, frequency and genetics of hereditary neurological diseases from the African continent. The autosomal dominant cerebellar ataxias (ADCAs), also known as the inherited spinocerebellar ataxias (SCAs), are a clinically and genetically heterogeneous group of progressive neurodegenerative disorders, characterized primarily by degeneration of the cerebellum, brainstem, and spinal cord. Clinically, the cerebellar syndrome (gait and limb ataxia, dysarthria, and nystagmus) is often associated with non-ataxia symptoms, such as pyramidal signs, ophthalmoplegia, visual disorders, seizures and peripheral neuropathy [2–4]. Phenotypes of different SCAs often overlap; therefore, clinical diagnosis remains a challenge.

More than 40 genes have been found to be associated with the SCAs, with SCA44 recently identified [5]. The most common types of SCAs worldwide include SCA1, SCA2, SCA3, SCA6, SCA7, and dentatorubral-pallidoluysian atrophy (DRPLA), all caused by unstable trinucleotide repeat Cytosine-Adenine-Guanine (CAG) expansions in the coding region of the respective genes [3, 6]. The size of the CAG repeat and the age at onset are inversely correlated: the longer the repeat, the earlier the age at onset.

Few studies on the clinical spectrum, frequency, and genetics of the SCAs are available from the African continent [7, 8]. Established studies mostly concern the South African populations, where there is a high frequency of SCA1, SCA2 and SCA7 [9–12]. Moreover, the SCA7 mutation in South Africa (SA) has been found almost exclusively in families of Black ethnic origin [10–13]. There is no published data on the spectrum of the SCAs in the Zambian population.

The objective of this study was to describe the clinical phenotype in seven Zambian families with autosomal dominant SCA, and perform molecular analyses on a subset of these families in order to confirm clinical findings.

## Methods

### Patients and families

**Fig. 1:**
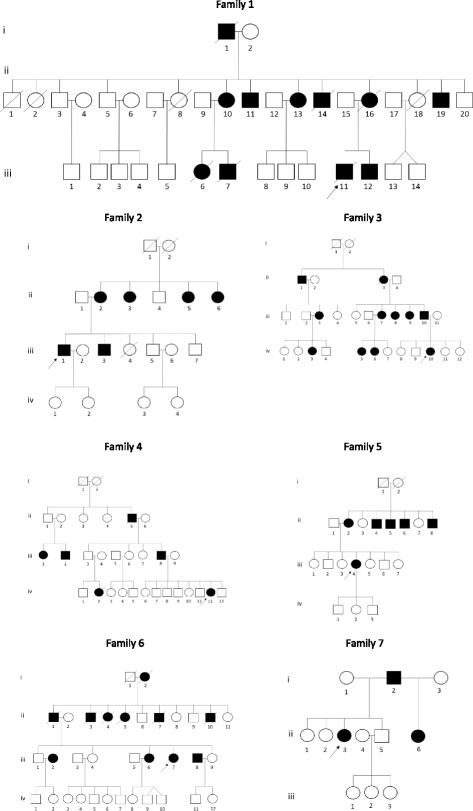
Family pedigrees of patients with spinocerebellar ataxia

The study was conducted at the University Teaching Hospital (UTH) in Lusaka, Zambia. UTH is the major teaching hospital for the University of Zambia School of Medicine (UNZA-SOM) and the main tertiary care center in Zambia. The Republic of Zambia is a landlocked country in the central part of southern Africa with a population of more than 15 million people. Although more than 70 different tribes are currently recognized, the Zambian population can be classified within the Niger-Kordofanian family, the largest of the four ethno-linguistic African macrofamilies [14]. Five ethnic Zambian families and two mixed (Zambian/Sierra-Leonean and Zambian/Zimbabwean) families with suspected SCA were examined between 2008 and 2015. Fig. 1 shows the pedigree of each family.

A neurologist (MA) examined probands, affected, and unaffected family members. The inclusion criteria included progressive cerebellar ataxia with or without associated extracerebellar symptoms, and a positive familial history compatible with autosomal dominant inheritance. Patients with ataxia possibly associated with abuse of alcohol or other substances and diseases, including HIV infection, were excluded. Ataxia was quantified with the Brief Ataxia Rating Scale (BARS) with a total rating score of 30, which is derived from the modified international ataxia rating scale [15]. Dementia was quantified with MMSE (Mini-mental state examination). The probands underwent routine laboratory testing and brain CT and/or MRI. Molecular genetic testing for SCA types was performed on 3 families at the National Health Laboratory Service (NHLS) at Groote Schuur Hospital and the Division of Human Genetics, University of Cape Town (UCT), South Africa (SA). All probands and family members who provided DNA samples gave written informed consent, and the study was approved by the Biomedical Research Ethics Committee of the University of Zambia. At the University of Cape Town (UCT), ethical approval was granted by the institutional Human Research Ethics Committee (HREC REF 229/2010 and 217/2010, renewed annually).

### Genetic analysis

Genomic DNA was isolated from peripheral blood samples in the Department of Internal Medicine UNZA/UTH in Lusaka using standard protocols and shipped to the Division of Human Genetics, UCT. The National Health Laboratory Service (NHLS) at Groote Schuur Hospital in Cape Town is the only centre in southern Africa that offers molecular genetic testing for the polyglutamine SCAs (SCA1, SCA2, SCA3, SCA6, SCA7 and SCA17) since testing was initiated in 1987 [11]. The CAG repeat region within each causative gene for this study was amplified using a multiplex PCR method [16] or a triplet-primed PCR (TP PCR) assay [17], followed by capillary electrophoresis on the ABI 3100 Genetic Analyzer (Applied Biosystems). The sizes of the repeats were determined by comparison with the GeneScan 500 Rox Size Standard (Applied Biosystems). Where TP PCR was used, the CAG repeat genotype of the individual was denoted using N to indicate an allele within the unaffected range, and E to indicate a pathogenically expanded allele (Table 1). Each run included positive control samples of known CAG repeat length.

**Table 1:** Clinical features of examined patients with spinocerebellar ataxia

Family	Individual	AO examination	AO Onset	AO Death	Genotype (SCA7)	Clinical symptoms									
						Ataxia	Visual loss	Ptosis	Ophthalmoplegia	Bulbar syndrome	Dysarthria	Spasticity	Dementia	CT	MRI
1	i.1	71	59	73	NT	x	x	x	x	x	x	x	x	Brainstem & cerebellar atrophy	
	ii.10	51	Early 40’s	U	N/E (9/50)	x	x	x	x	x	x	x			Diffuse cerebral and cerebellar atrophy, mild brainstem and cortical atrophy
	ii.11	36	U	U	N/E (9/55)	x	x	x	x		x		x		Ponto-Cerebellar ND cortical atrophy
	ii.13	44	U	U	N/E (9/50)	x	x	x	x		x				Cerebellar and mild pontine and cortical atrophy
	ii.14		18	29	N/E	x	x			x	x				
	ii.16		29	36	NT	x	x	x	x	x	x	x	x	Mild cerebral and severe cerebellar atrophy	Mild cerebral and severe cerebellar atrophy
	ii.19	39	U	U	NT	x	x	x	x		x				Cerebellar atrophy and cortical atrophy.
	iii.11	12	12	U	10/56	x	x	x	x		x	x		Cerebellar atrophy	Cerebellar atrophy
	iii.12	20	17	U	9/47		x	x						Cerebellar atrophy	
2	iii.1	33	29	U	N/E	x	x		x		x		x		Severe diffuse cerebral and cerebellar atrophy
	iii.3	30	U	U	N/E (9/52)	x	x		x		x		x		Olivo-ponto-cerebellar and cortical atrophy
3	iii.11		U	U	NT	x	x		x						
	iv.10	26	16	U	NT	x	x	x					x		Mild cerebral and cerebellar atrophy
4	iii.8	68	60	U	N/E	x	x		x		x		x		Diffuse cerebral and cerebellar atrophy
	iv.12	25	16	U	N/E	x	x	x	x		x			Cerebellar and diffuse cerebral atrophy	Cerebellar and diffuse cerebral atrophy
5	ii.2		53	U	NT	x								Cerebellar atrophy	
	iii.4	36	28	U	NT	x			x		x		x	Mild cerebral and moderate cerebellar atrophy	
6	iii.7	25	19	U	NT	x					x		x		Cerebellar atrophy and mild pons atrophy
7	ii.3	31	23	U	NT	x					x				Mild cerebellar atrophy

*AO* Age of, *U* unknown, *NT* not tested, *N* indicates CAG allele within unaffected/normal range, *E* indicates CAG allele within pathogenic range, number of CAG repeats in brackets, if available

## Results

Seven families with autosomal dominant cerebellar ataxia were identified. Three families had molecularly confirmed SCA7. Another family had a clinical phenotype suggestive of SCA7 but did not undergo genetic testing. Three additional families were found to have clinical phenotypes associated with autosomal dominant SCA, but genetic material was not available for molecular confirmation.

Accessible family members in different generations who were available for examination manifested with  similar clinical phenotypes.

Clinical and radiological data of 19 patients with autosomal dominant spinocerebellar ataxia are presented in Table 1. The mean age at onset (32 ± 14, range 12-73) was not significantly different in males and females. All patients had progressive cerebellar ataxia. In 15 of the 19 patients ataxia was associated with decreased visual acuity, ptosis and ophthalmoplegia. Pigmentary macular degeneration was found in 8 of them, optic atrophy in 3 and both symptoms were evident in 4. Nine patients had symptoms of dementia. Six patients had tremor, dystonia and other extrapyramidal symptoms. Almost all patients had increased reflexes, but in 4 individuals it was associated with spasticity of the lower limbs and peripheral sensory neuropathy. Various degrees of cerebellar or olivo-ponto-cerebellar atrophy on CT or MRI was found in all 19 patients with additional cortical atrophy in ten. The pathogenic SCA7 CAG repeat ranges varied from 47 to 56 repeats.

### Family 1

**Fig. 2:**
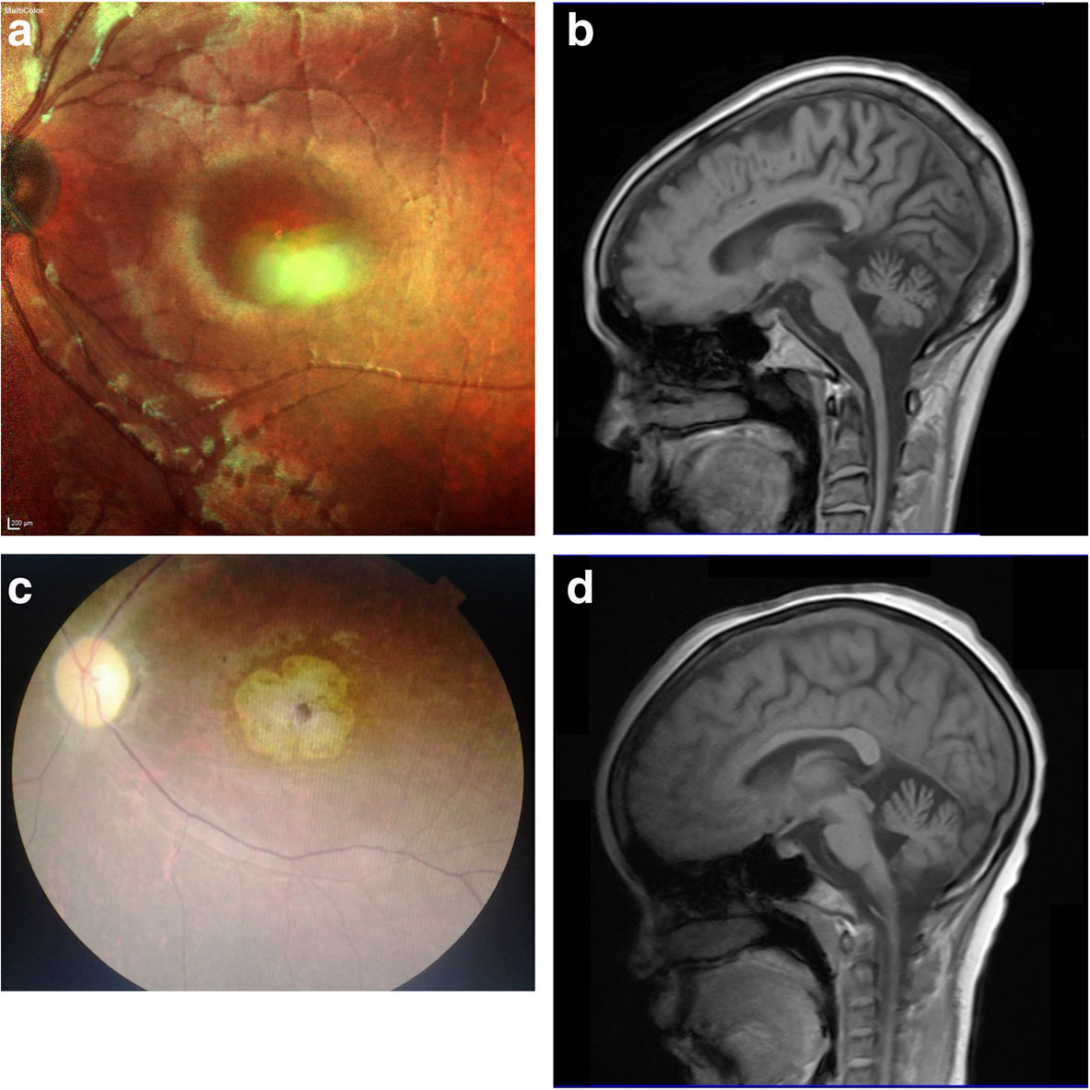
**a** Photograph of the right fundus of the proband’s brother of Family 1 (iii.12), showing macular degeneration. **b** T2-weighted MRI brain of patient ii.10 of Family 1 demonstrating diffuse cerebral and cerebellar atrophy, and mild brainstem atrophy. **c** Photograph of the right fundus of individual iv.10 of Family 3 showing macular degeneration and optic atrophy. **d** T2-weighted MRI brain of individual iv.10 of Family 3 showing mild cerebral and cerebellar atrophy

Family 1 was from the Bemba tribe in the Copperbelt (Kitwe) region. The proband (iii.11) was 14 years old at examination, and presented with progressive cerebellar symptoms and visual loss starting at 12 years. The general physical examination was normal. The neurological examination revealed severe gait ataxia, limbs ataxia, bilateral ptosis, bilateral pyramidal symptoms and external ophthalmoparesis. Sensation was normal. MMSE showed a mild mental impairment. Fundoscopy revealed bilateral macular degeneration. A CT scan of the brain revealed cerebellar atrophy, confirmed by MRI. The proband died at age 21 from pneumonia. The father of the proband didn’t have any neurological symptoms. The brother (iii.12) of the proband was asymptomatic up to 17 years old, but had developed visual problems several months before examination. The general physical examination was normal, but neurological examination revealed mild bilateral ptosis and depressed reflexes. Fundoscopy revealed bilateral macular degeneration (Fig. 2a).

The mother (ii.16) of the proband was one of 13 children. Five of her siblings had similar clinical features of progressive ataxia, visual loss, dysarthria and spasticity. Her symptoms started at age 29. She presented with progressive gait ataxia, blindness, bilateral ptosis and ophthalmoparesis, dysarthria, bulbar symptoms, dystonic movements of the limbs, spasticity and dementia. Fundoscopy revealed retinopathy along with optic atrophy. CT and MRI showed mild cerebral and severe cerebellar atrophy. She lost vision at age 32, and died at age 36. Molecular genetic analysis of the proband, his brother, and three maternal family members revealed *ataxin-7* repeats within the disease-causing range (Table 1). MRI brain of the affected aunts (ii.10, ii.13) and uncles (ii.11, ii.19) showed various degrees of olivo-ponto-cerebellar atrophy with cortical atrophy which progressed during 6 years of observation. Cortical atrophy was correlated with cognitive impairment. All of them had decreased visual acuity associated with varying degrees of macular pigmentary changes on ophthalmoscopy. Clinical features of the extended family are presented in Table 1.

### Family 2

Family 2 was from the town of Mongu in western Zambia (Lozi tribe). The proband (iii.1) was a 33-year-old male who presented with progressive ataxia and poor vision over a period of 4 years. The neurological examination revealed severe gait ataxia, symmetrical ataxia of limbs, dysmetria, dysarthria, slowing of eye movements, bilateral pyramidal symptoms, peripheral sensory neuropathy, dementia and euphoria. Ophthalmic examination revealed decreased visual acuity in 85% and bilateral pigmentary macular degeneration along with optic atrophy. MRI scan of the brain showed severe diffuse cerebral and pontocerebellar atrophy. Cortical atrophy was correlated with significant cognitive decline. He had 2 daughters, both in good health. The brother (iii.3) of the proband was 30 years old. All neurological, ophthalmologic and imaging findings were similar to the proband. The mother (ii.2) of the proband and her 3 sisters had severe balance and vision problems, which started in their mid-50s and 60s. These relatives were not examined. Molecular genetic testing confirmed the diagnosis of SCA7 in the proband and his brother.

### Family 3

Family 3 was from the city of Chipata in eastern Zambia. The proband (iv.10) was a 26-year old female who presented with progressive reduction in visual acuity and difficulty with walking due to severe imbalance, which started 4 years prior to examination. She had severe bilateral and symmetrical ataxia of lower limbs and gait, but mild ataxia in the arms. She had mild bilateral ptosis without ophthalmoparesis, and slowing of eye movements. Very mild fasciculation of the tongue and bilateral pyramidal signs (very high tendon reflexes, bilateral clonus and Babinski sign), as well as mild distal hypoesthesia of lower limbs. Fundoscopy revealed bilateral macular degeneration and optic atrophy (Fig. 2c). MRI of the brain showed mild cerebral and cerebellar atrophy (Fig. 2d). The proband had 2 healthy children. The mother of the proband (iii.11), from the Ngoni tribe, did not have any neurological symptoms. Her other four children did not have any history of imbalance or poor vision. The mother’s brothers, sisters and their children were healthy.

The father (iii.10) of the proband, from the Kunda tribe, was affected with the same condition. At age 56 he started progressive reduction in visual acuity (over 4 years) and difficulty in walking due to severe imbalance. Of his four sisters, 3 (iii.7, iii.8 and iii.9) had similar symptoms such as reduced visual acuity, severe gait ataxia, and loss of memory. These symptoms started at age 49, 50 and 52. Clinical features of extended family members are presented in Table 1. No genetic material was available for molecular confirmation of diagnosis.

### Family 4

Family 4 was from the Nyanja tribe. The proband (iv.12) was a 25-year old female who reported onset of gait difficulties at age 16, which was later associated with progressive loss of vision. She also complained of dysphagia. The general physical examination was normal. Her neurological examination revealed severe gait ataxia, limbs ataxia, dysarthria, bilateral ptosis and external ophthalmoparesis, along with bulbar and bilateral pyramidal symptoms. Sensation was normal. Her mental examination showed a moderate mental impairment. Fundoscopy revealed bilateral macular degeneration and optic atrophy. A CT and MRI scan of the brain revealed cerebellar and diffuse cerebral atrophy. Her father (iii.8) was examined at the age 68. His balance and visual problems started at age 60. He was unable to walk without assistance and nearly blind. Neurological examination was positive for cerebellar ataxia, macular retinopathy, ophthalmoparesis, slurred speech, bulbar and bilateral pyramidal symptoms. He had severe dementia. MRI of the brain revealed diffuse cerebral and cerebellar atrophy. His father (ii.5) (grandfather of the proband), who was Zimbabwean had developed gait ataxia, poor vision and blurring speech at the age of 70. Two cousins of the proband’s father had similar symptoms which started at ages 32 and 35. The proband’s cousin (iv.2) developed gait disturbances and poor vision at the age 19 and died at the age of 28. A diagnosis of SCA7 was molecularly confirmed in the proband and his father (Table 1).

### Family 5

Family 5 was from the Ngoni tribe. The proband (iii.4) was a 36-year old female, presenting with an 8-year history of gradual progression in difficulty walking and slurred speech. Neurological examination demonstrated gait and limbs ataxia, dysarthria, slow ocular saccades, mild ophthalmoparesis, bilateral distal sensory neuropathy, hands tremor and mild dementia. Fundoscopy did not show retinopathy or maculopathy. CT of the brain showed mild cerebral and moderate cerebellar atrophy. Her three children and siblings were asymptomatic.

The mother (ii.2) of the proband had limb ataxia from age 53, and had developed peripheral motor and sensory neuropathy by the age of 58. Her fundoscopy was unremarkable, CT of the brain showed cerebellar atrophy. Her four brothers were affected with similar symptoms, with unsteadiness starting after age 40. Affected siblings of the mother had not been examined. No genetic material was available for molecular confirmation of diagnosis.

### Family 6

Family 6 was from Livingstone in southwestern Zambia (Lozi tribe). The proband (iii.7) was a 25-year old female who presented with a 6-year history of progressive unsteadiness and hand shaking. The general physical examination was normal. Neurological examination revealed severe limb, truncal, and gait ataxia. There was also dysarthria, and a persistent head and hand resting tremor. She had mild dementia, slow saccadic eye movements and reduced deep tendon reflexes. Sensation was intact. Fundoscopy was unremarkable. MRI of the brain showed cerebellar atrophy and mild pons atrophy. Her two sisters (iii.2, iii.6) had developed limb and gait ataxia and resting tremor of hands since ages 23 and 26. The proband’s mother (ii.2) did not report any neurological symptoms and was clinically healthy. The proband’s father (ii.1), paternal grandmother (i.2), uncles (ii.3, ii.7. ii.10) and aunts (ii.4, ii.5) had similar symptoms. Ataxia, dystonia, tremor of hands, saccadic eye movements and symptoms of peripheral neuropathy were the main symptoms of affected members of the family, which started at age 68 (ii.1), 73 (i.2), 66 (ii.3), 64 (ii.7), 63 (ii.10), 67 (ii.4) and 65 (ii.5). None had ophthalmological problems. No genetic material was available for molecular confirmation of diagnosis.

### Family 7

The proband (ii.3) was a 31-year old female who presented with an 8-year history of progressive unsteadiness and slurred speech. The general physical examination was normal. Her neurological examination found mild cerebellar gait and limb ataxia, dysarthria, and slow eye movements, along with decreased vibration sense and reflexes. She had mild memory loss, and fundoscopy was unremarkable. MRI of the brain showed mild cerebellar atrophy. The patient refused permission to collect blood for DNA analysis. The proband’s mother was Zambian. Her father (i.2) was Sierra-Leonean, and developed balance issues in his mid-50s. Another daughter (ii.6), born from a Sierra Leonean marriage, developed unsteady gait in her early 30s and slurred speech by the age 36. No genetic material was available for molecular confirmation of diagnosis.

## Discussion and conclusions

Five affected families were ethnic Zambians from various tribes (including Bemba, Nyanja, Lozi, and Kunda), originating from different regions of the country (Eastern, Western and Central province). The Kunda tribe are found in Eastern Province of Zambia. And interestingly, this tribe is also found in Zimbabwe and Mozambique. Two families were of mixed ancestry (one Zambian and Zimbabwean, one Zambian and Sierra-Leonean).  In all families, the disease had an autosomal dominant pattern of inheritance across multiple generations.

The ADCAs are rare disorders with an estimated global prevalence of 2-7 per 100 000 [2]. The frequency of these disorders varies widely in different populations and geographical regions. Studies conducted worldwide show that in many countries SCA3 is the most common ataxia, followed by SCA2 and SCA6. A relatively high frequency of SCA3 has been seen in Brazil [18], Portugal [19], China [20], Japan [21], Thailand [22], the Netherlands, and Germany [3]; but is lower in France, Canada, and USA; and rare in Italy, Norway and India [3]. A high prevalence of SCA2 was found in Cuba [23] and Mexico [24, 25]. Four of the seven Zambian families studied showed phenotypes typical of SCA7, including the characteristic presence of retinopathy. SCA7 is caused by the expansion of a CAG repeat tract in the *ataxin-7* gene (ATXN7), which translates into a polyglutamine-expanded protein. The mutated protein, causes neuronal loss in the cerebellum, brainstem and retina [26, 27]. Pathogenic, expanded alleles typically contain between 38 and 70 repeats, but extreme expansions – up to 460 CAG – have been documented [28]. SCA7 is a relatively rare form of ADCA, but has variable frequencies in different regions [10, 11, 25, 29, 30]. The risk of CAG triplet expansion into the disease-associated range is frequently associated with haplotypes, making individuals with certain haplotypes at lower or higher risk for the disease [31]. The relative frequency of SCA7 is considerably higher in certain regions of the world, including Mexico, South Africa and Scandinavia, and is associated with local founder effects in those areas [12, 24, 30, 32].

Reports on SCA7 in the African continent are scarce, although there are isolated reports from Algeria, Angola, Cape Verde, Liberia, Mali, Morocco, South Africa and Tunisia [9, 13, 18, 26, 33, 34]. SA has one of the highest frequencies of SCA7 in the world. Moreover, the SCA7 mutations in SA have only been found in families of Black ethnic origin [10–13]. A haplotype study in 13 families from the indigenous Black African population of SA provided evidence for a SCA7 founder effect [11, 12]. Importantly, haplotype studies based on Families 1 and 4 have confirmed that this SA SCA7 haplotype extends to northern Namibia and Zambia [35]. Considering the geographical distribution of these families, it’s feasible to hypothesize that the South African SCA7 founder effect extends to additional neighbouring countries such as Botswana and Zimbabwe. Population-based haplotype studies may give important insights into the origins of pathogenic mutations, and can assist in the design of therapeutic interventions, such as RNA interference or CRISPR based modalities.

The main phenotypes in families 1, 2, 3 and 4 were gait and limb ataxia, dysarthria, visual loss, ptosis, ophthalmoparesis or ophthalmoplegia, pyramidal tract signs and dementia. The combination of these symptoms with retinal degeneration and cerebellar atrophy are in line with SCA7, which was molecularly confirmed families 1, 2 and 4. All families displayed anticipation of both onset age and rate of disease progression. There was marked anticipation in age of onset in Family 1 (Table 1). Moreover, the rate of the disease progression was significant in the proband and his mother, with the mother of the proband dying 7 years after disease onset and the proband dying 9 years after disease onset. The proband, his mother, grandfather and aunt also displayed symptoms of spasticity. SCA7 and hereditary spasticity may co-exist within this family.

In the proband and his affected brother in family 2, cerebellar ataxia correlated with cerebellar atrophy, decreased visual acuity with macular degeneration and cortical degeneration with cognitive impairment. This suggests that motor systems could degenerate in parallel with visual systems and cognitive decline with cortical degeneration, although it is difficult to determine whether the degeneration in motor, visual and cortical systems is caused by the same mechanisms.

In general, SCA7 anticipation is commonly observed through paternal transmission. Analysis of the dynamic mutation in the SCA7 gene may show marked parental effects on CAG repeat transmission. In families 1, 2 and 3, the third generation had maternal transmission, which, may be an important factor for anticipation of age of onset and the rate of disease progression. Genetic anticipation through the maternal transmission has previously been reported in Japanese families [36]. In family 4, the disease had paternal transmission and the affected grandfather of the proband was Zimbabwean. The clinical characteristics of Zambian families with SCA7 are similar to families described in Africa and other countries. The presence of progressive cerebellar ataxia, macular degeneration or optic atrophy, slow eye movements or ophthalmoparesis and pyramidal symptoms associated with cerebellar atrophy in brain imaging suggests that clinical picture of SCA7 is relatively homogenous.

The combination of cerebellar symptoms with slow saccadic eye movements, peripheral neuropathy, dementia and tremor suggest phenotypic presentation of ADCA in families 5, 6 and 7. All three families displayed marked anticipation of onset age. These features suggest that these families are likely to be affected with a different type of SCA possibly caused by a polyglutamine repeat expansion (SCA1, 2, 3, 6, 12 and 17), but genetic material was not available for molecular analysis. Family 7 was of mixed ancestry. The affected Sierra-Leonean father of the proband had affected daughters from Sierra-Leonean and Zambian marriages, suggesting that the origin of the mutation in this family may be Sierra Leonean.

In conclusion, we have shown that SCA is evident in ethnic Zambian families. These findings imply that all ethnic Zambian patients with suspected inherited ataxia should have molecular genetic testing. Furthermore, these families should be counselled with their relatives who are at risk of inheriting the altered gene. Further clinical and genetic studies of the prevalence of the spinocerebellar ataxias in a larger cohort of patients may help shed light on the natural history and origin of these disorders in the Zambian population.

## Abbreviations

ADCA: Autosomal dominant cerebellar ataxias
BARS: Brief Ataxia Rating Scale
CAG: Cytosine-Adenine-Guanine
CT: Computer tomography
DRPLA: Dentatorubral-pallidoluysian atrophy
HREC: Human Research Ethics Committee
MMSE: Mini-mental state examination
MRI: Magnetic resonance imaging
NHLS: National Health Laboratory Service
SA: South Africa
SCA: Spinocerebellar ataxia
UCT: University of Cape Town
UNZA-SOM: University of Zambia School of Medicine
UTH: University Teaching Hospital

## References

[1] Campbell MC, Tishkoff SA (2008). African genetic diversity: implications for human demographic history, modern human origins, and complex disease mapping. Annu Rev Genomics Hum Genet.

[2] Schöls L, Bauer P, Schmidt T, Schulte T, Riess O. Review Autosomal dominant cerebellar ataxias : clinical features, genetics, and pathogenesis. Lancet. 2004;3:291–304. doi:10.1016/S1474-4422(04)00737-9. 10.1016/S1474-4422(04)00737-9 15099544

[3] Durr A (2010). Autosomal dominant cerebellar ataxias: polyglutamine expansions and beyond. Lancet Neurol.

[4] Rossi M, Perez-Lloret S, Doldan L (2014). Autosomal dominant cerebellar ataxias: a systematic review of clinical features. Eur J Neurol.

[5] Watson LM, Bamber E, Schnekenberg RP, Williams J, Bettencourt C, Lickiss J, Fawcett K, Clokie S, Wallis Y, Clouston P, Sims D, Houlden H, Becker EBE. Németh AH. Dominant mutations in GRM1 cause spinocerebellar ataxia type 44. Am J Hum Genet. 2017;101(3):451–8. doi:10.1016/j.ajhg.2017.08.005. 10.1016/j.ajhg.2017.08.005 PMC5591020 28886343

[6] Matilla-Dueñas A, Corral-Juan M, Volpini V, Sanchez I. The spinocerebellar ataxias: clinical aspects and molecular genetics. Neurodegen Dis. 2012. p. 351–74. doi:10.1007/978-1-4614-0653-2_27. 10.1007/978-1-4614-0653-2_27 22411256

[7] Sridharan R, Radhakrishnan K, Ashok PP, Mousa ME (1985). Prevalence and pattern of spinocerebellar degenerations in Northeastern Libya. Brain.

[8] Osuntokun B, Adeuja A, Schoenberg B (1987). Neurological disorders in Nigerian Africans: a community-based study. Acta Neurol Scand.

[9] Modi G, Modi M, Martinus I, Rodda J, Saffer D. The clinical and genetic characteristics of spinocerebellar ataxia type 7 (SCA 7) in three Black South African families. Acta Neurol Scand. 2000;101(3):177–82. doi:10.1034/j.1600-0404.2000.101003177.x. 10.1034/j.1600-0404.2000.101003177.x 10705940

[10] Bryer A (2003). The hereditary adult-onset ataxias in South Africa. J Neurol Sci.

[11] Smith DC, Bryer A, Watson LM, Greenberg LJ (2012). Inherited polyglutamine spinocerebellar ataxias in South Africa. S Afr Med J.

[12] Greenberg J, Solomon GAE, Vorster AA, Heckmann J, Bryer A (2006). Origin of the SCA7 gene mutation in South Africa: implications for molecular diagnostics. Clin Genet.

[13] Smith DC, Greenberg LJ, Bryer A (2016). The hereditary ataxias: where are we now? Four decades of local research. S Afr Med J.

[14] Tishkoff SA, Reed FA, Friedlaender FR (2009). The genetic structure and history of Africans and African Americans. Science.

[15] Schmahmann JD, Gardner R, MacMore J, Vangel MG (2009). Development of a brief ataxia rating scale (BARS) based on a modified form of the ICARS. Mov Disord.

[16] Dorschner MO, Barden D, Stephens K. Diagnosis of five spinocerebellar ataxia disorders by multiplex amplification and capillary electrophoresis. J Mol Diagn. 2002;4(2):108–13. doi:10.1016/S1525-1578(10)60689-7. 10.1016/S1525-1578(10)60689-7 PMC1906987 11986402

[17] Smith DC, Esterhuizen A, Greenberg J (2013). Caution regarding the interpretation of homoallelism in polyglutamine multiplex assays: a recommendation for confirmatory testing of homozygous alleles. J Mol Diagn.

[18] Teive H, Munhoz R, Arruda W (2012). Spinocerebellar ataxias – genotype-phenotype correlations in 104 Brazilian families. Clinics.

[19] Vale J, Bugalho P, Silveira I (2010). Autosomal dominant cerebellar ataxia: frequency analysis and clinical characterization of 45 families from Portugal. Eur J Neurol.

[20] Tang B, Liu C, Shen L, et al. Frequency of SCA1, SCA2, SCA3/MJD, SCA6, SCA7, and DRPLA CAG trinucleotide repeat expansion in patients with hereditary spinocerebellar ataxia from Chinese kindreds. Arch Neurol. 2000;57(4):540–4. doi:10.1001/archneur.57.4.540. 10.1001/archneur.57.4.540 10768629

[21] Maruyama H, Izumi Y, Morino H (2002). Difference in disease-free survival curve and regional distribution according to subtype of spinocerebellar ataxia: a study of 1,286 Japanese patients. Am J Med Genet.

[22] Boonkongchuen P, Pongpakdee S, Jindahra P (2014). Clinical analysis of adult-onset spinocerebellar ataxias in Thailand. BMC Neurol.

[23] Velázquez-Pérez L, Rodríguez-Labrada R, García-Rodríguez JC, Almaguer-Mederos LE, Cruz-Mariño T, Laffita-Mesa JM (2011). A comprehensive review of spinocerebellar ataxia type 2 in Cuba. Cerebellum.

[24] Alonso E, Martínez-Ruano L, De Biase I (2007). Distinct distribution of autosomal dominant spinocerebellar ataxia in the Mexican population. Mov Disord.

[25] García-Velázquez LE, Canizales-Quinteros S, Romero-Hidalgo S (2013). Founder effect and ancestral origin of the spinocerebellar ataxia type 7 (SCA7) mutation in Mexican families. Neurogenetics.

[26] Martin J, Van Regemorter N, Del-Favero J, Löfgren A, Van Broeckhoven C. Spinocerebellar ataxia type 7 (SCA7) - correlations between phenotype and genotype in one large Belgian family. J Neurol Sci. 1999;168(1):37–46. doi:10.1016/S0022-510X(99)00176-8. 10.1016/s0022-510x(99)00176-8 10500272

[27] David G, Dürr A, Stevanin G, et al. Molecular and clinical correlations in autosomal dominant cerebellar ataxia with progressive macular dystrophy (SCA7). Hum Mol Genet. 1998;7:165–70. doi:10.1093/hmg/7.2.165. 10.1093/hmg/7.2.165 9425222

[28] Sequeiros J, Martindale J, Seneca S (2010). EMQN best practice guidelines for molecular genetic testing of SCAs. Eur J Hum Genet.

[29] Storey E, du Sart D, Shaw JH (2000). Frequency of spinocerebellar ataxia types 1, 2, 3, 6, and 7 in Australian patients with spinocerebellar ataxia. Am J Med Genet.

[30] Jonasson J, Juvonen V, Sistonen P (2000). Evidence for a common spinocerebellar ataxia type 7 (SCA7) founder mutation in Scandinavia. Eur J Hum Genet.

[31] Pringsheim T, Fiest K, Jette N (2014). The international incidence and prevalence of neurologic conditions: how common are they?. Neurology.

[32] Magaña J, Tapia-Guerrero Y, Velázquez-Pérez L, et al. Analysis of CAG repeats in five SCA loci in Mexican population: epidemiological evidence of a SCA7 founder effect. Clin Genet. 2013:1–7. doi:10.1111/cge.12114. 10.1111/cge.12114 23368522

[33] Stevanin G, David G, Dürr A (1999). Multiple origins of the spinocerebellar ataxia 7 (SCA7) mutation revealed by linkage disequilibrium studies with closely flanking markers, including an intragenic polymorphism (G3145TG/A3145TG). Eur J Hum Genet.

[34] Traoré M, Coulibaly T, Meilleur KG (2011). Clinical and genetic analysis of spinocerebellar ataxia in Mali. Eur J Neurol.

[35] Smith DC, Atadzhanov M, Mwaba M, Greenberg LJ (2015). Evidence for a common founder effect amongst South African and Zambian individuals with spinocerebellar ataxia type 7. J Neurol Sci.

[36] Katagiri S, Hayashi T, Takeuchi T, Yamada H, Gekka T, Kawabe K, Kurita A, Tsuneoka H. Somatic instability of expanded CAG repeats of ATXN7 in Japanese patients with spinocerebellar ataxia type 7. Doc Ophthalmol. 2015;130(3):189–95. doi:10.1007/s10633-015-9488-8. 10.1007/s10633-015-9488-8 25643591

